# Large-Conductance Calcium-Activated Potassium Channels in Glomerulus: From Cell Signal Integration to Disease

**DOI:** 10.3389/fphys.2016.00248

**Published:** 2016-06-21

**Authors:** Jie Tao, Zhen Lan, Yunman Wang, Hongya Hei, Lulu Tian, Wanma Pan, Xuemei Zhang, Wen Peng

**Affiliations:** ^1^Department of Nephrology and Central Laboratory, Putuo Hospital, Shanghai University of Traditional Chinese MedicineShanghai, China; ^2^Department of Pharmacology, School of Pharmacy, Fudan UniversityShanghai, China

**Keywords:** BK channels, podocytes, mesangial cells, β subunits, glomerular filtration

## Abstract

Large-conductance calcium-activated potassium (BK) channels are currently considered as vital players in a variety of renal physiological processes. In podocytes, BK channels become active in response to stimuli that increase local cytosolic Ca^2+^, possibly secondary to activation of slit diaphragm TRPC6 channels by chemical or mechanical stimuli. Insulin increases filtration barrier permeability through mobilization of BK channels. In mesangial cells, BK channels co-expressed with β1 subunits act as a major component of the counteractive response to contraction in order to regulate glomerular filtration. This review aims to highlight recent discoveries on the localization, physiological and pathological roles of BK channels in glomerulus.

## Introduction

BK channels, also known as Maxi-K because of the large single-channel conductance (>200 pS in 100 mmol/L symmetrical K^+^), constitute a particular family of ion channels, which couple intracellular chemical signaling to membrane electrical signaling (Rothberg, 2012; Contreras et al., 2013). BK channels distributing in excitable as well as non-excitable cells are considered as key participants in a lot of physiological functions, including regulating neuronal firing, smooth muscle tone, endocrine cell secretion, cell proliferation and migration (Contreras et al., 2013).

Functional BK channels are homotetramers made up of pore-forming α subunits which are encoded by a single gene (*Slo1* or *Kcnma1*). Distinct from homologous voltage-gated K^+^ (Kv) channels, BK α subunit possesses an additional hydrophobic segments containing a N-terminal transmembrane helix (S0) at the extracellular side of the plasma membrane as well as a long cytosolic C-terminal (S7–S10) which is comprised of two RCK (regulator of K^+^ conductance) domains and calcium bowl where putative Ca^2+^-binding sites reside (Yuan et al., 2010). Cooperating with tissue-specific auxiliary β (β1-4) and γ (γ1-4) subunits, BK channels are divided into several subfamilies and play specific roles in physiological actions of different organizations. The auxiliary subunits are responsible for modulating the kinetic behavior and Ca^2+^ sensitivity of BK as well as pharmacological responses to BK specific modulators (Tao et al., 2011; Contreras et al., 2013; Table 1).

**Table 1:** **BK auxiliary subunits in glomerulus**.

**Subunit**	**Gene symbol**	**Tissue (s)**	**Functional effect**
β1	*KCNMB1*	Mesangial cell; rat podocyte	Facilitates voltage-sensor activation; IbTX insensitive; ChTX sensitive (Ma et al., 2005; Piwkowska et al., 2015)
β2	*KCNMB2*	Rat podocyte	Inactivation; IbTX sensitive; ChTX insensitive (Piwkowska et al., 2015)
β3	*KCNMB3*	Human podocyte	Inactivation; IbTX sensitive; ChTX insensitive (Morton et al., 2004)
β4	*KCNMB4*	Rat and human podocyte	Facilitates voltage-sensor activation; IbTX insensitive; ChTX insensitive; MarTX sensitive (Morton et al., 2004; Kim et al., 2008; Piwkowska et al., 2015)
γ1	*LRRC26*	Arterial smooth muscle cell	Facilitates voltage-sensor activation; IbTX sensitive (Contreras et al., 2013)
γ2	*LRRC52*	Kidney	Facilitates voltage-sensor activation (Contreras et al., 2013)
γ3	*LRRC55*	Not reported	Facilitates voltage-sensor activation (Contreras et al., 2013)
γ4	*LRRC38*	Not reported	Facilitates voltage-sensor activation (Contreras et al., 2013)

In addition to their physiological functions, BK channels are also involved in a series of diseases such as hypertension, epilepsy, cancer and so on (Contreras et al., 2013). Recently, BK channel subtypes have been discovered in podocytes and mesangial cells (Morton et al., 2004; Ma et al., 2005; Piwkowska et al., 2015). In this review, we summarize the latest progress on the expression, function and regulatory mechanism of BK channel subtypes in glomerulus and evaluate the feasibility of therapeutical BK-specific ligands for chronic kidney disease (CKD).

## BK channels in glomerular podocytes

Podocytes, one of highly specialized cells encircling glomerular capillaries, extend the foot processes (or pedicels) forming finger-like projections, referred to as the slit diaphragm, interdigitate with that of neighboring cells. The sophisticated structure comprises an important component of the glomerular-filtration-barrier. The slit diaphragm containing cytoskeletal proteins and ion channels, such as nephrin, podocin, Neph1, F-actin, TRPC6, and BK channels, regulates podocyte function and cell body in response to fluid tresses (Dryer and Reiser, 2010; Lennon et al., 2014; Piwkowska et al., 2015).

### Molecular properties of podocyte BK

BK channels and β subunits have been successively discovered expressed in mouse, rat and human podocytes (Morton et al., 2004; Dryer and Reiser, 2010; Piwkowska et al., 2015). Podocyte BK channels with slow kinetics and large conductance were observed in whole-cell as well as single-channel recordings readily, which was partially inhibited by iberiotoxin (IbTX), but completely blocked by paxilline and penetrem A (Morton et al., 2004; Kim et al., 2008). Iberiotoxin-insensitive BK (α+β4) is probably the main subtype in podocytes supported by the evidence above. It has been suggested that podocyte BK channels could be activated in response to membrane stretch at the positive potentials and high level of intracellular Ca^2+^. The opening probability of BK grew fourfold by applying modest negative pressure or decreasing the tonicity of bath solution (Morton et al., 2004). However, it is still unclear the mechanism underlying the activation of BK under physiological conditions, which is possibly associated with SD proteins of podocyte.

### Interaction of podocyte BK with SD proteins and TRPC6

**Figure 1:**
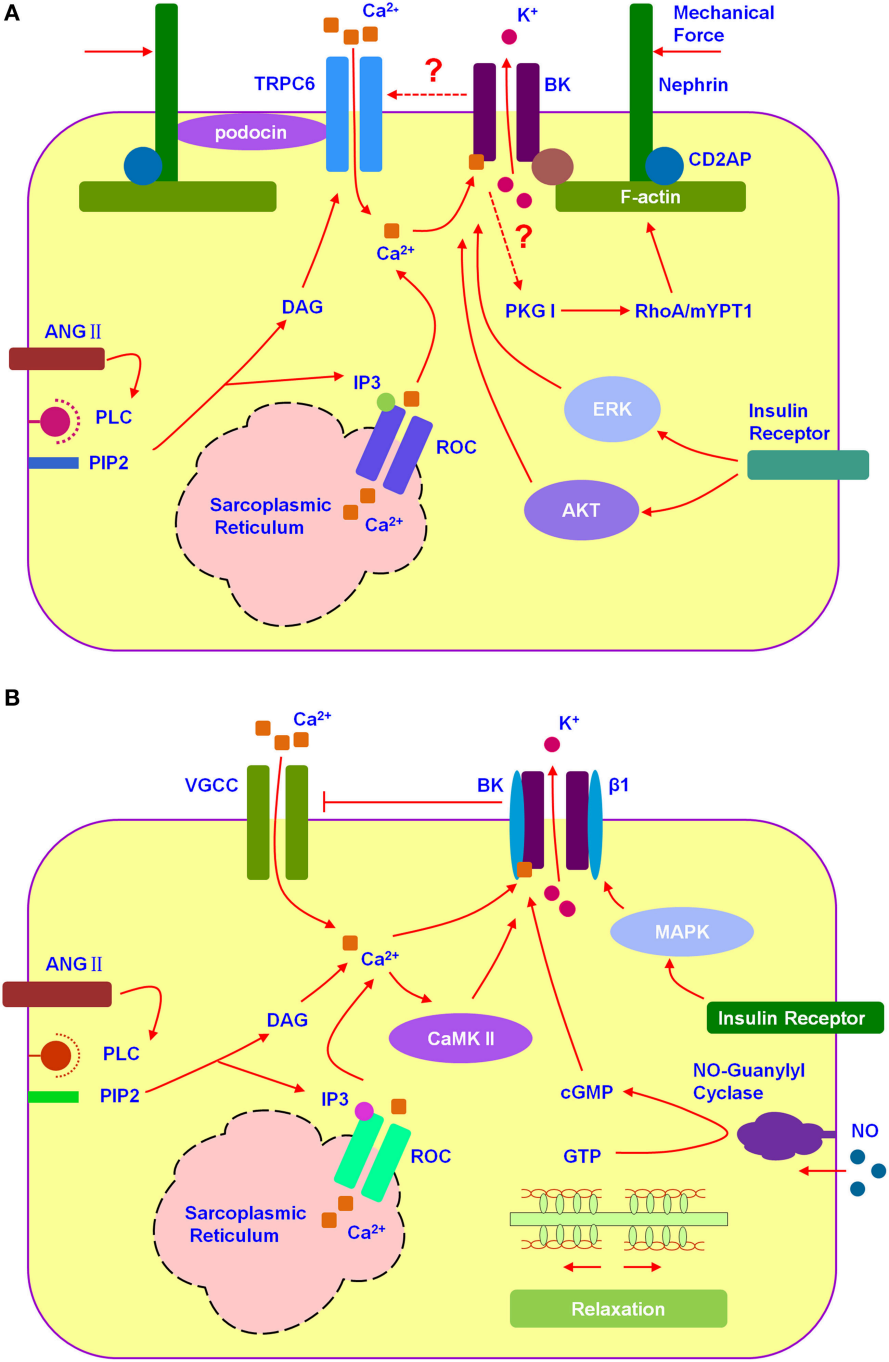
**BK channels participated in the signal integration of podocytes and mesangial cells. (A)** Hypothesized relation among TRPC6, BK, and SDs in podocyte. Podocyte BK participated in the cytoskeleton-related signal integration. **(B)** Hypothesized relation among VGCC and BK in mesangial cell. Mesangial BK participated in the signal integration of mesangial cell relaxion.

SD proteins (Figure 1A), interacting with BK C-terminal, include nephrin (Kim et al., 2008), Neph1 (Kim et al., 2009b), MAGI-1 (Ridgway et al., 2009), and synaptopodin (Kim et al., 2010). Down-regulating the expression of nephrin or synaptopodin decreases the number of functional surface BK channels in mouse podocytes (Kim et al., 2008, 2010). Co-expression of synaptopodin or nephrin with *Slo1* increases surface expression of BK channels in HEK293T, which is the possible mechanism underlying the increment of BK protein expression during the maturity of podocytes (Yang et al., 2013). On the contrary, down-regulation of MAGI-1 mediates an increment in functional surface expression of BK in podocytes, co-expression of MAGI-1 suppresses surface expression of BK on the HEK293T cells (Ridgway et al., 2009). The situation with Neph1 is complicated because its regulation on BK surface expression depends on the cell type (Kim et al., 2009b). However, none of the proteins mentioned above facilitate the activation of BK channels. Thus, it is strongly suggested two possibilities for the activation of BK in non-excitable podocytes. One is SD proteins interacting with β subunits, the other is a stretch-dependent increment in intercellular Ca^2+^ concentration, for example BK interacting with mechanosensitive TRPC6 of which the stretch-evoked activation was markly regulated by podocin (Anderson et al., 2013).

TRPC6 (Figure 1A), one of the non-selective cation channels, has been demonstrated co-expressing with BK channels and promoting its surface expression in podocytes (Kim et al., 2009a). TRPC6 permeates Ca^2+^ depending on the membrane hyperpolarization (Estacion et al., 2006). BK could be activated by TRPC6-induced calcium influx as well as membrane depolarization. Then, BK-caused membrane hyperpolarization provides positive feedback to TRPC6. Gain-of-function mutations in TRPC6 have been discovered, which mediate inherited glomerular disease such as focal segmental glomerulosclerosis (FSGS) (Reiser et al., 2005; Winn et al., 2005). Because of the close structure between each TRPCs member, there is still no specific blockers for TRPC6 (Dryer and Reiser, 2010). BK blockers seem to be an ideal way to control the glomerular sclerosis induced by TRPC6 hyperfunction. Among all, martentoxin, a selective blocker for podocyte BK (α+β4), is worthy of in-depth study for anti-FSGS (Tao et al., 2014).

### Regulation of hormones and pathological environment on podocyte BK

Growing evidences have suggested that several hormones (Figure 1A), such as angiotensin II and insulin, as well as pathological environment regulating BK expression and function playing important roles in podocyte injury (Kim and Dryer, 2011; Gao et al., 2015; Piwkowska et al., 2015). Angiotensin II (Ang II) which could induce the oxidative stress and podocyte death not only inhibits the current amplitude of Podocyte BK, but also facilitates the BK activation (Gao et al., 2015). Insulin increases cell surface expression of podocyte BK channels, with accompanied by a corresponding increase in the current density, via ERK (extracellular signal-regulated kinase) and AKT (PKB, protein kinase B) signaling cascades. While, high glucose treatment decreases the number of functional surface BK channels and nephrin as well as abolishes the stimulatory effects of insulin on BK (Kim and Dryer, 2011). Podocyte BK is also considered as a key player mediating insulin-increased filtration barrier permeability along with PKGI-dependent transepithelial albumin flux through participating in the disruption of the actin cytoskeleton induced by insulin. IbTX blocked insulin-induced disruption of the actin cytoskeleton as well as inhibited the phosphorylation of PKG target proteins, RhoA and MYPT1 (Piwkowska et al., 2015). The exposure of podocytes to hypoxia environment caused an obvious reduction in BK currents and shifted BK activation range toward more depolarized potential and slowed its activation kinetics via increased BK β4-subunits expression (Zhang et al., 2012).

## BK channels in glomerular mesangial cells

Mesangial cells (MC) in glomerulus have been proved participating in many physiological activities, such as producing growth factors, forming mesangial matrix as a structural support for capillaries and modulating glomerular hemodynamics through contractile properties. Confronted with glomerular injury induced by inflammation or hypertension, MCs often changes its phenotype as myofibroblasts expressing α-smooth muscle actin or interstitial collagens in addition to normal matrix constituents (Ma et al., 2005).

### Molecular properties and cell function of mesangial BK

MCs have a lot of properties in common with smooth muscle cells, mainly expressing BK as well as VGCCs (voltage-gated calcium channels) (Ma et al., 2005; Figure 1B). Different from BK in podocyte, BK in MCs acts as a “brake” on Ca^2+^ signal. The activation of BK promotes membrane hyperpolarization, which provides negative feedback to VGCCs and the counteractive response to contraction of mesangial cell (Stockand and Sansom, 1996). Mesangial BK was composed by α and β1 subunit which has been shown to increase the sensitivity of BK to intercellular Ca^2+^ significantly (Kudlacek et al., 2003). MCs relax to elevate glomerular filtration rate (GFR) when the body is volume expanded. BK-β1 knockout mice have been observed a normal GFR under basal conditions, but they fail to elevate their GFR to the same extent as wild-type mice upon volume expansion (Pluznick et al., 2003). It has been demonstrated that BK-β1 gain-of-function variant-Glu65Lys could obviously influence GFR. From clinical investigation, the 65Lys carriers exhibit not only elevated baseline GFR, but also decline GFR more rapidly in CKD (Chen et al., 2010). Evidences above suggest that the BK (α+β1) is a key regulator for the tone of MCs. BK openers depending on the presence of β1 subunits have the potential to be a useful scaffold in the development of drugs for regulating GFR, for example newly discovered GoSlo-SR family (Large et al., 2015).

Besides VGCCs, store-operated Ca^2+^ (SOC) channels, including Orai1, are also functional expressed in MCs (Sours-Brothers et al., 2009; Wu et al., 2015). Orai1 could negatively regulate the expression of extracellular matrix protein, such as fibronectin and collagen IV, as well as mesangial expansion in the renal cortex (Wu et al., 2015). In recent years, store-operated Ca^2+^ channels have been reported to be associated with BK in many cells (Gueguinou et al., 2014). In contrast to VGCCs, Mesangial BK probably provides positive feedback to SOC channels and plays a reno-protective role in glomerular disease. However, there is no direct evidence that SOC channels are combined with BK in mesangial cells so far.

### Regulation of signaling molecules on mesangial BK

Signaling molecules activated BK could also be considered as ideal drug targets for regulating GFR. In MCs, pathways including protein kinase, hormones and gases are involved in regulating BK function (Figure 1B). It has been proved through patch clamping that BK could be activated by cGMP and PKG as well as by soluble gas and guanylyl cyclase stimulators such as atrial natriuretic peptide (ANP) and nitric oxide (NO), respectively (Ma et al., 2005). Insulin activates mesangial BK and upregulates BK expression not only in cellular but also in plasma membrane via MAPK pathway (Foutz et al., 2008). Ca^2+^/calmodulin-dependent kinase II (CaMKII) acts as an endogenous agonist to expand the Ang II-induced activation of BK and cause a feedback to MCs contraction (Sansom et al., 2000).

## Concluding remarks and perspectives

In glomerular podocytes, BK channels, interacting with most SD proteins, participate in the increase of filtration barrier permeability induced by insulin as well as the remodeling pathway of cytoskeleton protein. β4 subunit is an important auxiliary subunit which controls the surface expression, dynamic characteristics and drug sensitivity of podocyte BK channels. BK channels, consisting of α and β4, could be used as a novel target for the treatment of glomerular disease. However, there are still a lot of mysteries of podocyte BK channels in suspense, (1) inactivation mechanism underlying the positive-feedback regulation of BK on Ca^2+^ signal in podocytes, (2) regulation of BK channel on the cytoskeleton of podocytes, (3) the reason why angiotensin II activated TRPC6, but inhibited the BK.

In glomerular mesangial cells, BK channels, co-expressed with β1, act as a major component of the counteractive response to contraction through negative-feedback regulating VGCCs. Therefore, specific openers of BK (α+β1) channels are expected to become the potential drugs for renal glomerular disease. It is a pity that suitable openers for BK (α+β1) haven't been found so far. Perhaps, agonists for cGMP and CaMKII pathway will become another way to solve this difficulty.

At last, because BK channels are widely expressed in most cells and tissues, the drugs designed for BK-related disease should be pay more attention to tissue-specific and BK subtype-specific properties in order to avoid the safety issues.

## Author contributions

Conceived the paper: XZ and WeP. Wrote the paper: JT, ZL, and YW. Drew the figure: JT. Revised the draft: HH, LT, and WaP.

## Funding

This work was supported by National Science Foundation of China (No.81370979 and 81573478), Shanghai Science and Technology Innovation Grant (No.14140903202), Key Medical Discipline project of Shanghai Municipal Health Bureau (No.ZK2012A34), Innovation Program of Shanghai Municipal Education Commission (No.15ZZ063), Shanghai Municipal Commission of Health and Family Planning Fund for Young Scholars (No. 20134050) and Research Project of Putuo Hospital, Shanghai University of Traditional Chinese Medicine, China (No. 2014YJ002).

### Conflict of interest statement

The authors declare that the research was conducted in the absence of any commercial or financial relationships that could be construed as a potential conflict of interest.

## Abbreviations

BK: Large-conductance calcium-activated potassium channels
K_v_: voltage-gated K^+^ channels
CKD: chronic kidney disease
HEK: human embryonic kidney
SD: slit diaphragm
IbTX: iberiotoxin
TRPC6: transient receptor potential canonical 6
FSGS: focal segmental glomerulosclerosis
Ang II: Angiotensin II
ERK: extracellular signal-regulated kinase
PKB: protein kinase B
PKG: cGMP-dependent protein kinase
MYPT1: myosin phosphatase target subunit 1
MC: Mesangial cells
VGCCs: voltage-gated Ca^2+^ channels
GFR: glomerular filtration rate
SOC: store-operated Ca^2+^ channels
ANP: atrial natriuretic peptide
NO: nitric oxide
MAPK: mitogen-activated protein kinase
CaMKII: calmodulin-dependent kinase II.
